# Challenges, Advances and Opportunities in Exploring Natural Products to Control Arboviral Disease Vectors

**DOI:** 10.3389/fchem.2021.779049

**Published:** 2021-11-15

**Authors:** Daniel P. Demarque, Laila S. Espindola

**Affiliations:** ^1^ Laboratory of Pharmacognosy, Department of Pharmacy, Faculty of Health Sciences, University of Brasilia, Brasilia, Brazil; ^2^ Laboratory of Pharmacognosy, Department of Pharmacy, School of Pharmaceutical Sciences, University of São Paulo, São Paulo, Brazil

**Keywords:** *Aedes aegypti*, insecticides, natural products, drug discovery, arboviral vectors, dengue

## Abstract

Natural products constitute an important source of molecules for product development. However, despite numerous reports of compounds and active extracts from biodiversity, poor and developing countries continue to suffer with endemic diseases caused by arboviral vectors, including dengue, Zika, chikungunya and urban yellow fever. Vector control remains the most efficient disease prevention strategy. Wide and prolonged use of insecticides has resulted in vector resistance, making the search for new chemical prototypes imperative. Considering the potential of natural products chemistry for developing natural products-based products, including insecticides, this contribution discusses the general aspects and specific characteristics involved in the development of drug leads for vector control. Throughout this work, we highlight the obstacles that need to be overcome in order for natural products compounds to be considered promising prototypes. Moreover, we analyze the bottlenecks that should be addressed, together with potential strategies, to rationalize and improve the efficiency of the drug discovery process.

## Introduction

Natural products (NP) are widely recognized as an important source of molecules for the discovery of useful chemicals, with the Food and Drug Administration (FDA) reporting that NP/NP-derivatives account for approximately one third of all drugs approved (Patridge et al., 2016). The multifaceted NP chemical space can be valuable either as a direct source of molecules or lead structures which may be used as starting structures for further optimization.

Naturally-sourced products have not only been exploited in healthcare and agrochemical applications, but also as insecticides with the potential to contain endemic arboviral diseases, caused by arthropod-borne viruses. Dengue, chikungunya, Zika and urban yellow fever are among the viruses transmitted by the *Aedes aegypti* mosquito. The most prevalent in South America is dengue, which has been reported since the 1980s (Guzman et al., 2010). Chikungunya and Zika emerged as public health concerns associated with neurological manifestations including encephalitis, meningitis and congenital malformations such as microcephaly in newborns (Christo, 2015). In 2016, Brazil experienced a Zika virus epidemic with 205,578 cases, alerting the public health system of the need to increase efforts to address *Aedes* reproduction (Lowe et al., 2018).

Arboviral diseases are included in the neglected diseases group as they cause morbidity and mortality in countries with environmental conditions favorable to mosquito proliferation, predominantly poor or developing countries (Oliveira et al., 2017). As such, research and development funding that would normally come from the private sector in these countries is reduced, leaving the public sector, which is susceptible to political decisions not always favorable to science, to provide financial support. Another limiting factor is the domestic unavailability of chemical products which are often subject to importation issues.

The fundamental strategy that public health systems employ to avoid arboviral diseases is vector control. However, mosquito adaptation and proliferation in urban areas remains a major health concern in many countries. Since the beginning of the 20^th^ century, chemicals have been used to control mosquitos, either as repellents, larvicides or insecticides (Ghosh et al., 2012; Pavela et al., 2019). Vaccination represents a promising strategy with the first dengue vaccine developed by Sanofi Pasteur (Dengvaxia^®^), released in 2015 for people from 9 to 45 years old. However, in 2017, it was restricted for people who had previously been infected by the virus after severe cases dengue were reported (Thomas and Yoon, 2019). A vaccine for the four serotypes with different formulae is being developed by Instituto Butantan (Brazil) in partnership with the NIH (National Institutes of Health, United States), and is currently in phase III trials (Galula et al., 2019). Therefore, the demand for new arbovirus vector control strategies is constant, either to minimize dependence on specific insecticide groups or to provide alternatives in cases of vector resistance usually due to indiscriminate insecticide use.

Research into naturally-sourced secondary metabolites is not only a way of preserving biodiversity, but also a means of protecting its biotechnological arsenal with the view to developing its scientific, social and environmental potential. The Convention on Biological Diversity (CBD) seeks to guarantee the sustainable use and conservation of biodiversity. If the results of your research can inspire a company to make a profit, the benefits can be shared and converted to ensure conservation, sustainability and a return on investment for research.

Despite numerous scientific publications reporting plant extracts/molecules with activity against arboviral vectors, botanical insecticides only constitute a small portion of the market market (Cantrell et al., 2012; Silvério et al., 2020; Valli et al., 2021). This discrepancy raises several questions: (i) Why are there so many active extracts in the literature, but few products? (ii) Are NPs (especially from plants) still a potential source for developing insecticides? (iii) What are the NPs development issues and the potential strategies to overcome the bottlenecks?

Considering that mosquito vector control constitutes the most effective preventive strategy to tackle arboviral diseases, our research group, together with the Ministry of Health, created the ArboControl Brasil project in 2016. The aim of this cross-functional network of national and international researchers is to identify and develop prototypes for alternative insecticides. With the knowledge acquired, this contribution addresses the aforementioned questions by examining both the general aspects of NP drug development and the specific characteristics to obtain drug leads in vector control. Moreover, we consider the bottlenecks that must be overcome and potential strategies to streamline the drug discovery process.

## Success of Insecticides From Natural Sources

### Why Use Natural Products as Insecticides?

Several compounds have been obtained from nature and used directly as insecticides or lead compounds. One such example is in chemical ecology, with plant secondary metabolites affording protection against insect attack. These compounds are exploited in insecticide applications (Kumar, 2012; Miresmailli and Isman, 2014). In addition, interest in NP insecticides is enhanced because they are potentially less harmful, less toxic to non-target organisms and naturally biodegradable (Walia et al., 2017). In theory, these characteristics make them more environmentally friendly than synthetic products. Interestingly, some of these insect-active compounds are present in the human diet, which is also supportive of their safety (Chaithong et al., 2006).

### Examples of Natural Products as Insecticides

**FIGURE 1:**
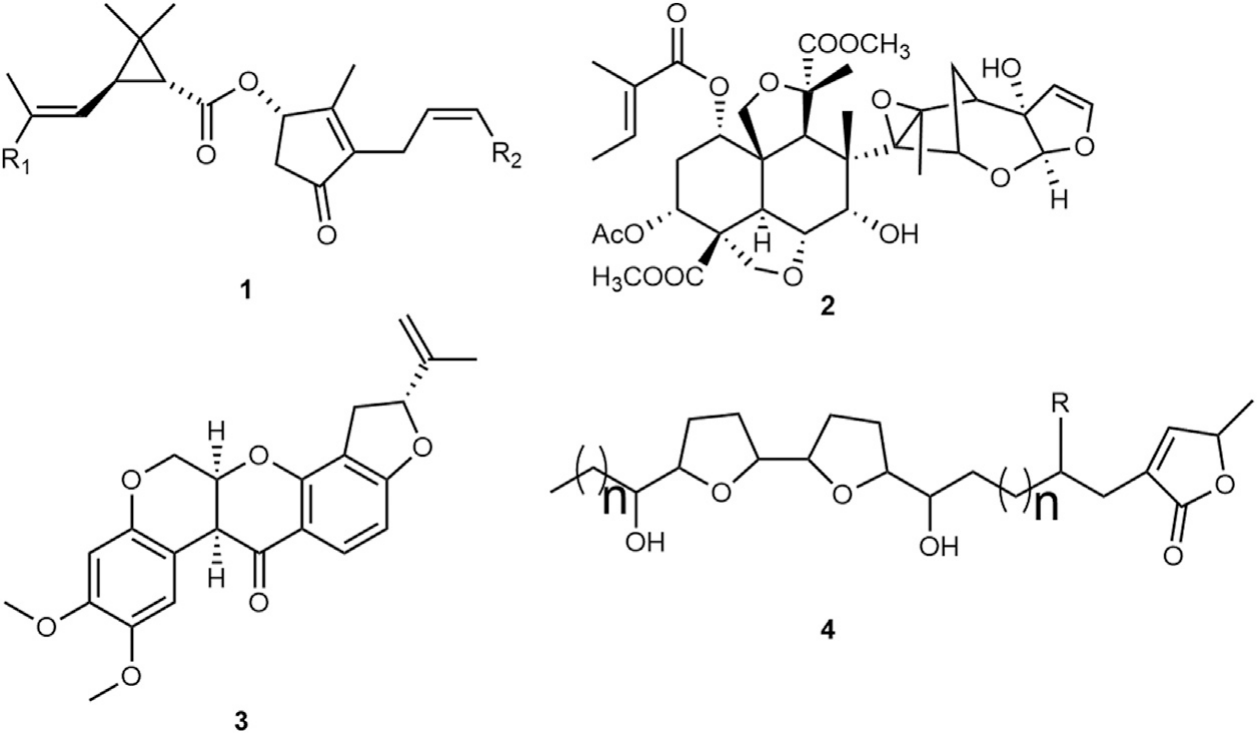
Molecules isolated from plants used as insecticides: pyrethrins 1; azadirachtin 2; rotenone 3, and acetogenins 4.

Natural insecticides are mainly sourced from plants and microorganisms. *Bacillus* thuringiensis is the most well-known microbial pesticide, with the activity of this bacteria attributed to proteins expressed by the Cry genes (El-kersh et al., 2014). Insecticidal plant compounds are exemplified in the scientific literature (Shaalan et al., 2005; Kishore et al., 2014; Suroowan et al., 2016). The pyrethrins 1, compounds initially obtained from *Chrysanthemum cinerariifolium* (Trevir.) Vis. (syn. *Tanacetum cinerariifolium* (Trevir.) Sch. Bip.) (Asteraceae) flowers, are an early example of vector control which inspired the development of synthetic analogs such as bioremethrin and tetramethrin, together with other pyrethroids, constituting the most commercially successful conventional insecticide classes (Figure 1). These compounds modulate the sodium channel in the insect nervous system, with the LC_50_ in *Aedes* spp. larvae ranging from 0.001 to 0.02 ppm, depending on the compound, formulation and/or strain susceptibility (Vontas et al., 2012; Smith et al., 2016).

One of the most recently studied plants in insecticide development is *Azadirachta indica* A. Juss. (Meliaceae), commonly known as neem, the seeds of which are used to extract azadirachtin 2, a potent and complex antifeedant tetranortriterpenoid. In addition, azadirachtin interferes with growth, moulting, reproduction and cellular processes (Isman, 2020). The LC_50_ is between 0.1–10 ppm depending on the mosquito species (Mordue(Luntz) and Nisbet, 2000), with the application cost almost three times that of the comparable synthetic insecticides (Shah et al., 2019).

Rotenone 3 is a lipophilic isoflavone first obtained from *Derris* and *Lonchocarpus* species (Heinz et al., 2017). Although this compound is a potent mitochondrial respiratory chain complex I inhibitor (LC_50_ ∼1.2 ppm) (He et al., 1997), it is nowadays considered inappropriate for vector control because of its extreme toxicity to fish (Isman, 2017).

Acetogenins 4, with a similar mechanism of action, are polyketide-derived fatty acid derivatives containing between 35 and 39 carbons, tetrahydrofuran rings inserted into its long-chain and a lactone ring. More than 400 structures have been identified in this class. Activity is structurally related, whereby compounds possessing bis-tetrahydrofuran rings and three hydroxyl groups have LC_50_ values ranging from 0.01 to 1 ppm (He et al., 1997).

The aforementioned examples, together with numerous others, highlight the potential of natural products in providing useful molecules to develop insecticides or lead structures that can be modified in order to provide better characteristics (Siegwart et al., 2015; Amoabeng et al., 2019; Isman, 2020; Silvério et al., 2020; Valli et al., 2021).

### How Is the Potency of an Extract/Compound Classified?

A common difficulty encountered by researchers developing NP screening is the classification of bioactive samples and the establishment of the adequate potency to be considered as a potential molecular starting point. The potency should be taken into account in conjunction with several other aspects, namely toxicity/selectivity, ease and cost of obtention, and structural complexity. An extract or compound that is not particularly potent, but has other potential characteristics, should be considered as a promising agent.

In any case, compounds active at low concentrations are advantageous. Some authors state that a standard screening concentration of ∼1,000 ppm (or µg/ml) which reduces 90% larval growth should be considered as relatively potent (Isman and Paluch, 2011). Regarding the aforementioned examples and the commercially available products, our research group considers an extract with an LC_50_ of ∼100 ppm as an appropriate starting point and compounds with an LC_50_ ≤ 10 ppm candidates for prototype development.

## Challenges for NP-Based Products

### Many Scientific Articles, Few Products

Natural products provide a viable option to keep pace with vector adaptability and associated resistance (Falkowski et al., 2020). Thus, efforts are ongoing in the exploration of biodiversity to source new extracts or active compounds. Despite several promising studies, few new naturally based insecticides capable of controlling arboviral vectors reach the marketplace (Marrone, 2019). This trend is mirrored in drug discovery as documented in a study which analyzed FDA-approved drugs from natural products and their derivatives. Patridge and co-workers (2016) reported a decrease in the contribution of plants in the discovery of new molecular entities, with a higher contribution provided by bacteria and fungi. This tendency is part of the drug discovery process itself, where few compounds reach the end of the pipeline due to problems relating to toxicity, potency, costs and many others. For unaltered NP compounds, access, supply, seasonal and environmental variations, absolute efficacy, speed of action, loss of source and physicochemical problems are specific and common bottlenecks that NP chemists are constantly trying to overcome.

Isman and Grieneisen (2014) analyzed the discrepancy between the number of scientific articles published and the botanical insecticides that reached the market. One point raised by the authors is the quality of the publications: many do not include chemical characterization and positive controls. This limited reproducibility contributes to explaining this inconsistency, underutilization of existing data and non-continuity of studies involving these plants. In addition, most investigations focused on the initial stages of chemical development (“basic research”) with few groups dedicating efforts to extraction, formulation development, scale-up and field application studies (“applied research”) (Isman, 2017).

### General Perspective

Scannell et al. (2012) discuss some primary factors that hinder innovation and decrease efficiency in the pharmaceutical and chemical industry. One important point raised is the advantages a new product must offer over the commonly used products. The competitive requirements for a new chemical are often higher, making development more challenging. From an economic perspective, the absence of differentiating attributes, such as being fast acting and having an appropriate toxicological/environmental profile, can reduce the potential of an extract/NP to enter the marketplace. Furthermore, the dominance and established production of an existing product guarantees a more affordable price, thereby making it more difficult for any new product to compete, even if it is more environmentally friendly.

Another factor hindering new product launches is the more stringent regulatory requirements. Agencies are demanding more comprehensive safety and efficacy tests than those previously applied to the numerous drugs or insecticides still in use today. While tighter regulations are necessary, this increased level of quality significantly augments research and development costs. For instance, a new agrochemical product takes 10–12 years to reach the market, with associated development costs estimated at US$ 286 million (Sparks and Lorsbach, 2017; Lorsbach et al., 2019).

In this context, toxicity prediction models should be continuously improved to minimize the false toxicity response. For example, many pharmaceutical drugs, including paracetamol, aspirin and penicillin, would fail to meet the current animal testing specifications. Paracetamol is toxic to dogs and cats, aspirin causes embryo toxicity in rats and rhesus monkeys, while penicillin is fatal to guinea pigs (Gayvert et al., 2016; Van Norman, 2019). Regarding plants, it could be cited that cinchona bark (*Cinchona* sp), the source of quinine, could cause blindness while ipecac (*Cephaelis ipecacuanha* (Brot.) A. Rich.), a source of emetine, can cause myocardial damage in certain test animals (Koppanyi and Avery, 1966; Knight, 2007). Several toxicity tests must be conducted to correctly predict toxicity during product development. Moreover, one test failure does not necessarily mean that a compound should be promptly discarded.

Regarding insecticides, herbicides and other pesticides, zebrafish (*Danio rerio*) constitutes a useful model to evaluate chemical toxicity in aquatic organisms, as it allows the assessment of different life stages, with several parameters at a low cost (Gonçalves et al., 2020; Schulte et al., 2021). Aerial organisms, such as honeybees and other pollinators, should also be considered in order to have a better understanding of ecotoxicity (Grillo et al., 2021).

Computational tools, such as the QSAR ToolBox developed by Organization for Economic Co-operation and Development (OECD), have been exploited to examine the structure-activity relationship and predict toxicity (Schultz et al., 2018). The use of computer software to make predictions based on descriptors extracted from existing similar structures, together with associated toxicity data, can streamline the decision-making process for candidates.

In addition to the aforementioned factors impacting innovation, other specific research challenges exist in the development of prototype insecticides against arboviral vectors, as discussed in the following sections.

## New Strategies to Identify Hit Structures

Biodiversity remains largely unexplored in terms of its chemical and biological activity. Pye et al. (2017) developed a retrospective analysis study of structures derived from NP in order to answer some key questions regarding how the structural novelty of NP has changed during past decades. The authors highlight that NP constitute a large unexplored chemical space and the use of technology/cutting edge solutions to avoid redundancy is imperative. Failure to adopt an innovative approach could result in the marginalization of NP research. In addition, numerous species are disappearing at an alarming rate as a result of uncontrolled anthropogenic activity which is often politically directed, especially in poor and developing countries. Therefore, it is concerning that active molecules that could contribute to arboviral disease vector control may also be lost.

**FIGURE 2:**
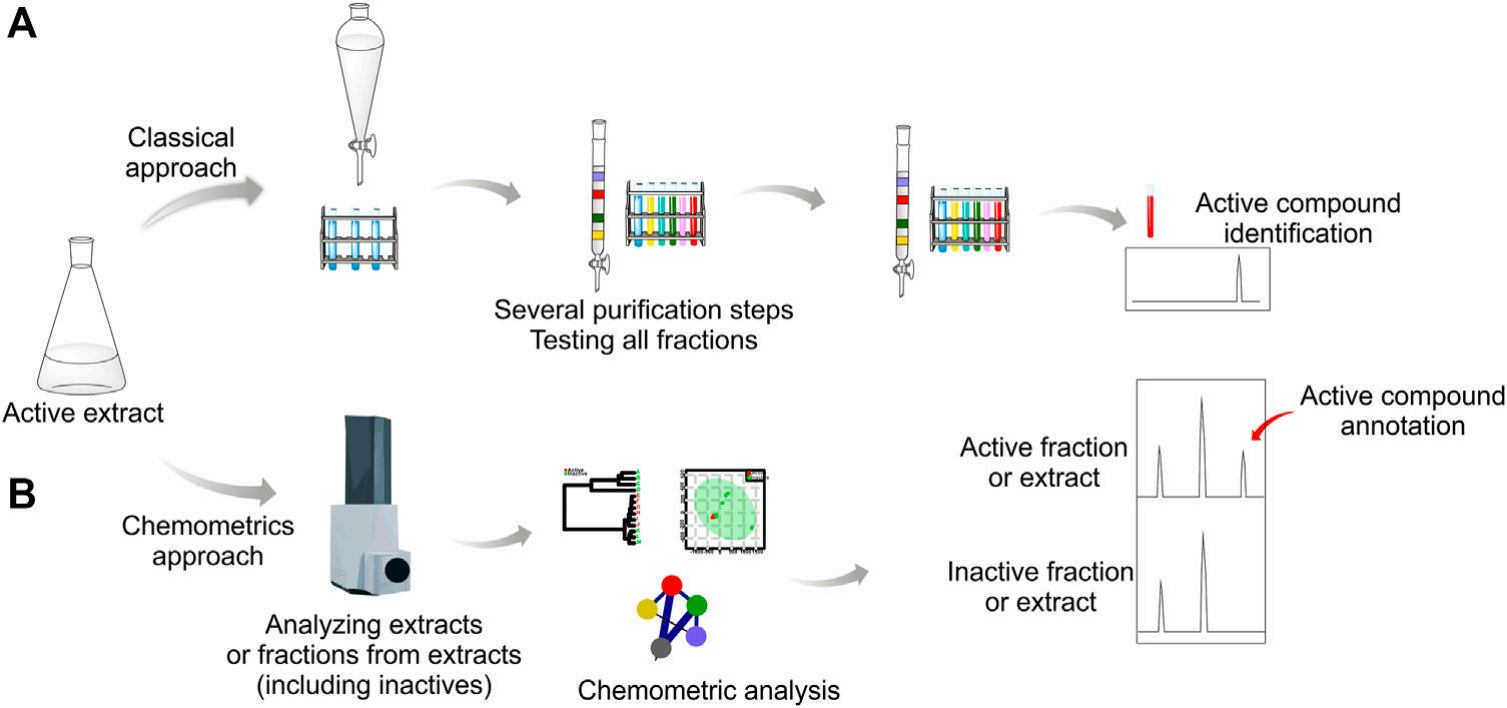
Comparison between classical **(A)** and chemometric **(B)** analysis approaches in the isolation of natural products. The classical approach relies on several purification steps until identifying the chemical(s) responsible for the activity. Chemometrics-based analysis involves the comparison of active and inactive samples to identify the presence of key compounds in active extracts that could be linked to the activity.

Advances in methodology and strategies have helped streamline the discovery process, effectively shortcutting the classical process in pinpointing molecules of interest in active samples (Aligiannis et al., 2016). This ‘metabolomics inspired’ strategy employs analytical chemistry techniques, together with computational and statistical treatments, to afford an overview of the metabolomic profile. Subsequent analysis promotes optimized data visualization (Figure 2B).

Chemometric techniques applied in a mass spectrometry-based metabolomics approach can be successfully employed to find the metabolic differences between active and inactive samples and, therefore, predict the potentially active compounds (Demarque et al., 2020). The strategy compares samples (extracts and/or fractions) from the same or closely related species (Figure 2B). Furthermore, this approach pinpoints the compound(s) of interest and permits dereplication, thus avoiding the re-isolation of known compounds when this is not the goal.

It is widely known that, for several reasons, many compounds in the initial phases of the pipeline will not result in a product. Successful research pipelines rely on a constant supply of new starting points to identify novel input chemicals. Metabolomics also contributes to the discovery of potentially new compounds to be used as hit structures. Searching chemical databases can reveal the absence of the pinpointed active compound, in which case isolation is required to confirm the hypothesis. This approach is considerably more rational (Morais et al., 2020).

## Challenges in NP Selectivity and Identifying New Targets

Insecticides can be classified according to their target entry mode and subsequent mechanism of action. An insecticide can enter as a stomach poison, contact poison or a fumigant (Rajashekar and Shivanandappa, 2017). They can affect/disrupt the nervous system, energy production, endocrine system, tegument (cuticle) development, and/or water balance. Rotenone (3) and acetogenins (4), for example, block energy production in insect mitochondria. However, as mammals share the same energy production system, 3 and 4 are also toxic for non-target animals (Rattan 2010).

Remarkably, around 94% of insecticides developed in the 1950s and 1960s were based on three main mechanisms of action: acetylcholinesterase (AChE) inhibition, gamma-aminobutyric acid (GABA)-gated chloride channel blocking and voltage-gated sodium channel modulation. During the 1990s, researchers argued that only a few receptors or biochemical processes would be susceptible for effective insect control. Different target sites have since been discovered. However, despite many of them being more selective, the number of new receptors or biochemical process targets remains limited, with related variations, for instance, different allosteric sites on the known receptors (Sparks et al., 2019).

Understanding insect receptor topology is an important approach in developing selective insecticides that act on different receptors. Finding alternative chemicals that can selectively kill insects usually requires knowledge of the ligand-binding domain, ligand-binding screening and/or virtual molecular docking. For instance, the development of selective neonicotinoid insecticides takes advantage of fundamental differences between the acetylcholine receptor in insects and mammals (Houchat et al., 2020). Essential oils have been extensively explored due to their potential selectivity, as most constituents act on the octopaminergic receptor, a non-mammalian target (Kostyukovsky et al., 2002).

Many unutilized receptors, biochemical processes and systems provide a source of innovative targets for more selective chemical development. One believes that many of the aforementioned targets remain sub-utilized as no chemical capable of acting on these targets has been developed, as exemplified in the case of neonicotinoids. In this context, the innovative structures of NP represent an opportunity to discover new pharmacophores.

## Strategies to Improve NP Activity

The initial screening strategy to preselect an extract active against *Ae. aegypti* larvae involves testing at a single concentration, standardized at 250 ppm by our research group. Many extracts exhibit low mortality in the initial test, however, a potent compound present in a low quantity may still be isolated. That said, it generally follows that a weak initial potency corresponds to little or no compound activity (de Sousa et al., 2020).

Positive initial screening results mostly relate to a high number of compounds acting with different mechanisms of action, accounting for why hundreds of plants are active against vectors. Moreover, detecting an extract which is active at the standardized concentration is relatively simple. Laboratory-based bioassays involve exposing insects (usually larvae) to compounds and observing the activity that is often a consequence of dietary imbalances: feeding deterrence/anorexia or impaired nutrient utilization. However, field test results are not guaranteed to mirror the lab-based results regarding this mechanism of action (Isman and Paluch, 2011).

**FIGURE 3:**
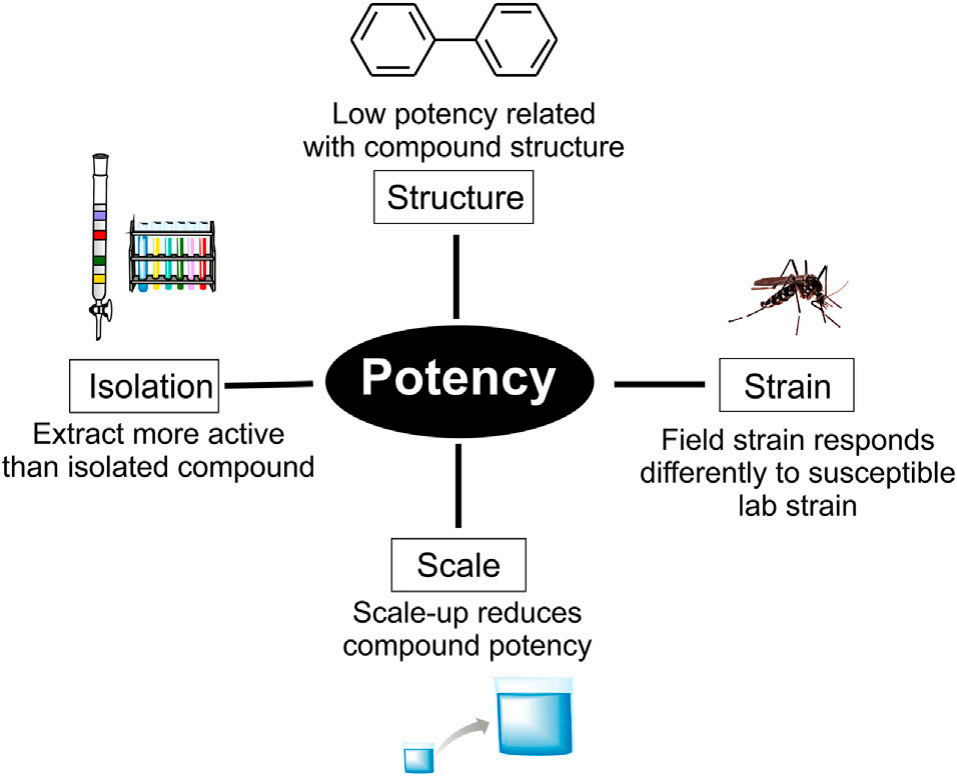
Common problems associated with the low potency of natural products.

The development of conventional insecticides focused on potent fast-acting agents. As previously mentioned, most of these successful agents affect the insect nervous system or other essential processes. NPs that act in a similar fashion are relatively rare, helping explain the contrasting high number of positive lab results and low number of leads in the pipeline (Figure 3).

For active isolated compounds, the physico-chemical properties, toxicity and selectivity can be improved by semi-synthesis. For example, phenylpropanoids can be modified in a similar manner to eugenol derivatives to increase potency (Barbosa et al., 2012). In some cases, larvicidal activity can be improved to the ppb level, as demonstrated in carnols from cashew nut shell liquid (11 ppm–2.3 ppb) (Paiva et al., 2017). Structure-activity relationships (SAR) are important when constructing a nature-inspired active compound to be used in a final product. In such cases, where compounds are moderately active, they constitute hits as opposed to lead drug candidates (Guantai and Chibale, 2011). This is also important to improve properties such as lipophilicity (logP). Due to plant vascular mobility, compounds usually have limited logP values (>3.5), which may limit the extent of penetration in insects (Bromilow et al., 1990; Jeschke, 2004).

Other causes of low potency can relate to test scale (Silva et al., 2020). Our group performs an initial small-scale test in 3 ml well plates in order to screen NP compounds frequently isolated in low quantities. The World Health Organization (WHO) recommends testing with cups (100–200 ml) which, due to the increased scale, decreases the activity of many compounds or alters solubility (Silva et al., 2020). Another important consideration is the *Ae. aegypti* strain used in initial laboratory studies, frequently the Rockefeller strain. Less promising activity can be observed in field-collected laboratory tests, small-scale field and field tests which involve exposure of native mosquito strains (Melo-Santos et al., 2010).

Loss of potency after isolation may also be attributed to synergic effects. Plants produce secondary metabolites which target pathogens through several combined mechanisms (Caesar and Cech, 2019), commonly the case for the insecticidal action of essential oils such as rosemary oil (Miresmailli et al., 2006; Tak and Isman, 2015, 2017). In this case, the use of extract standards should be considered (Demarque et al., 2015). Strategies that exploit synergic effects gather compounds in the same formulation, one such example is pyrethrin formulations with slow pyrethrin detoxification, piperonyl butoxide (PBO), N-octyl bicycloheptene dicarboximide (MGK 264), rotenone and ryania (Dimetry, 2014). Nanoparticles also offer an excellent alternative (Benelli, 2016).

## Strategies to Improve NP Sources

NP prototype development is hindered by the source itself, as native plants whose commercial interest has not yet led to their domestication, limits scale-up. Genetic variability, together with all climatic and environmental factors involved in secondary metabolite production, may also affect the presence of compounds of interest (Gobbo-Neto and Lopes, 2007; Gobbo-Neto et al., 2010; Yang et al., 2018). In addition, antifeedant molecule production may be triggered by the presence of predators, with intermittent production impacting collection.

All plant parts can be collected, with leaves more readily accessible. Roots and stems require more time and care during collection to avoid/minimize damage. It has been reported that the exposure of these two parts to soil pathogens, particularly their bark, account for the accumulation of important antifeedant/insecticide compounds as a defensive response to pathogen exposure (Da Costa et al., 2014).

Unnecessary plant part collection can be reduced by employing prior metabolomic analysis to detect the molecule of interest when the target is known. LC-MS/MS can perform specific searches using multiple reaction monitoring (MRM), in which the spectrometer generates a chromatogram when it detects the mass of the molecule of interest in the first stage (MS1) undergoing fragmentation, giving rise to a specific fragment ion in the second stage (MS2) (Demarque et al., 2016). This specific type of analysis is termed targeted metabolomics.

Another strategy, which is both environmentally and economically advantageous, is using abundant sources of raw material, such as industrial waste and commercialized ornamental or food plants (Singha and Chandra, 2011). Furthermore, the latter poses less risk to humans. Commercial compound screening constitutes another approach (Chen et al., 2008; Silva et al., 2020), by broadening the scope of known compounds (drug repurposing) with well documented toxicity, physico-chemical properties and obtention, thereby accelerating the product development pipeline.

## Strategies to Improve Insecticide Residuality

Residuality studies are mandatory in insecticide development. Biodegradation of plant insecticides limits their prolonged presence in the environment which is one of their main advantages (Schulte et al., 2021). There is a fine balance between insecticide efficacy over a defined period and biodegradation. Environmental persistence is undesirable, but necessary to guarantee residuality, facilitate the logistics of application, reduce costs (Isman, 2020) and above all successfully control the vector. Therefore, formulation studies are fundamental to ensure solubility in application preparation, stability during storage and in the target environment (particularly to rain/UV-light exposure), and compliance with health and safety regulations.

Strategies employed to improve formulation stability and effectiveness include nanoformulation and microencapsulation (Werdin González et al., 2014; Pessoa et al., 2018; Campos et al., 2020; Shahzad and Manzoor, 2021). The impregnation of zedoary oil in sand granules also remarkably prolonged activity from 5 days to 3 weeks (Champakaew et al., 2007; Sutthanont et al., 2019). In addition, the presence of antioxidants can prolong insecticide residuality as observed in pyrethrin formulations (Dimetry, 2014). Although it may seem a less problematic consideration, finding the right combination to maintain the active ingredient soluble and dispersible in water can prove difficult, as most actives are highly apolar compounds, exemplified by the aforementioned compounds.

## Summary and Outlook

Arboviral disease control is almost completely reliant on an insecticide-based strategy. Considering that commercially available insecticides persist in the environment, harm non-target organisms, promote resistance in mosquitoes, and pose long-term risks for humans and the environment, new strategies are clearly needed to control the *Ae. aegypti* vector. Genetically-modified mosquitoes can constitute part of a future alternative control strategy, although effectiveness is still being evaluated (Wilke et al., 2009; Waltz, 2021). Therefore, we highlight the importance of natural products as an important source of chemical prototypes for new product development.

**FIGURE 4:**
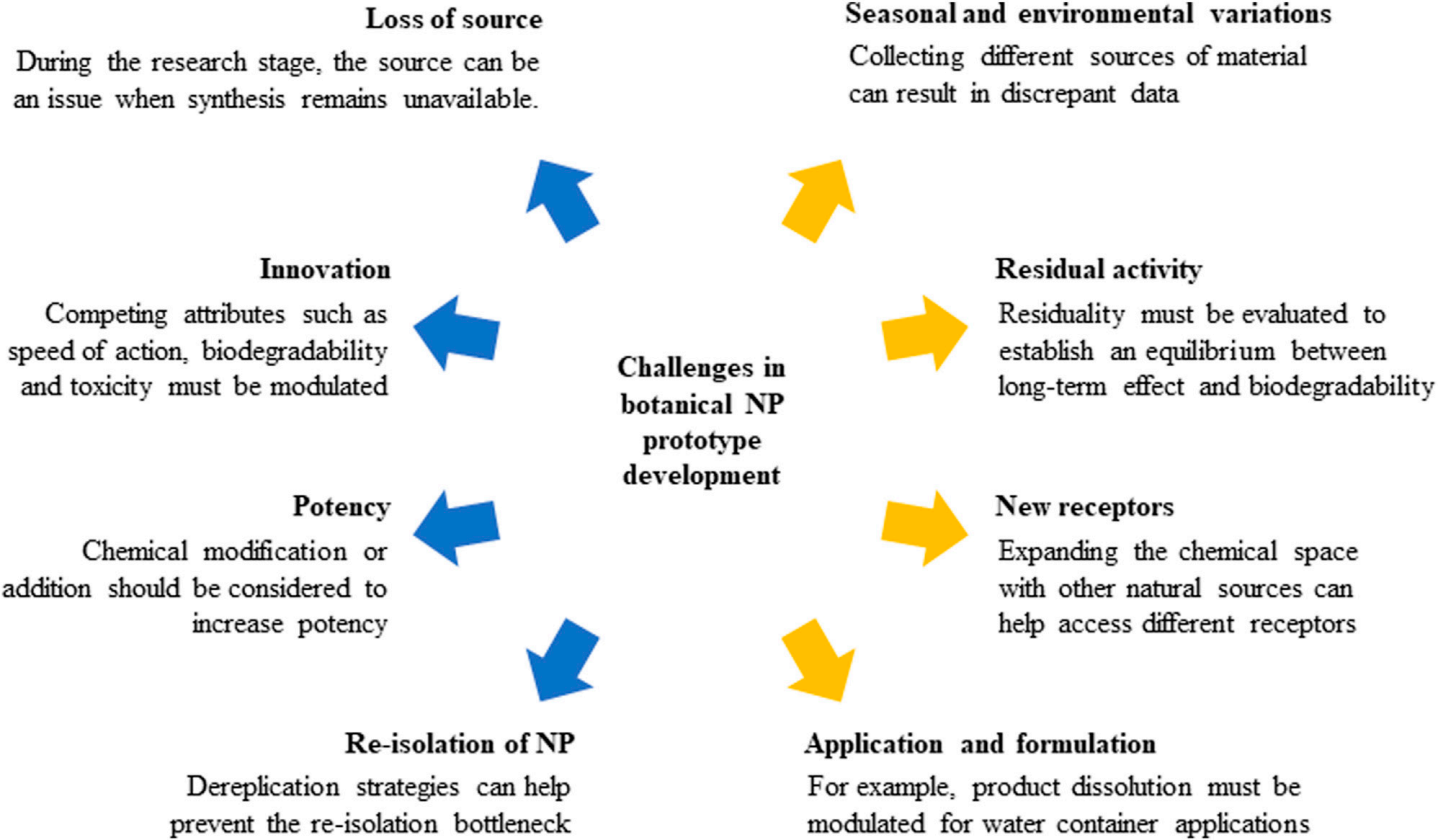
Bottlenecks that NP chemistry needs to address to develop agents against arbovirus vectors.

NP are responsible for ∼17% of the new insecticides listed by the International Standards Organization (ISO) over the last 28 years (Sparks et al., 2019). Despite this apparently low number, the impact of NP chemistry in new insecticide discovery is notorious in terms of the development of novel chemical leads and mechanisms of action. Herein we highlighted (i) the potential of NP as a source of compounds for new product development, together with (ii) the bottlenecks associated with NP-based insecticide development and (iii) the technological advances providing a more streamlined discovery strategy (Figure 4).

In summary, anthropogenic activities continue to foster arboviral vector proliferation and hamper disease control efforts. Multidisciplinary teams are essential to tackle the aforementioned significant challenges, exploit natural products to obtain prototypes and, most importantly, shorten the development time of novel insecticides.
